# Cluster Analysis of US COVID-19 Infected States for Vaccine Distribution

**DOI:** 10.3390/healthcare10071235

**Published:** 2022-07-02

**Authors:** Dong-Her Shih, Pai-Ling Shih, Ting-Wei Wu, Cheng-Jung Li, Ming-Hung Shih

**Affiliations:** 1 Department of Information Management, National Yunlin University of Science and Technology, Douliu 64002, Taiwan; wutingw@yuntech.edu.tw (T.-W.W.); abc867123@gmail.com (C.-J.L.); 2 Department of Information Management, National Chung Cheng University, Chiayi 621301, Taiwan; d08530003@ccu.edu.tw; 3 Department of Electrical and Computer Engineering, Iowa State University, 2520 Osborn Drive, Ames, IA 50011, USA; mshih@iastate.edu

**Keywords:** COVID-19, clustering analysis, classification validation, vaccine distribution, machine learning

## Abstract

Since December 2019, COVID-19 has been raging worldwide. To prevent the spread of COVID-19 infection, many countries have proposed epidemic prevention policies and quickly administered vaccines, However, under facing a shortage of vaccines, the United States did not put forward effective epidemic prevention policies in time to prevent the infection from expanding, resulting in the epidemic in the United States becoming more and more serious. Through “The COVID Tracking Project”, this study collects medical indicators for each state in the United States from 2020 to 2021, and through feature selection, each state is clustered according to the epidemic’s severity. Furthermore, through the confusion matrix of the classifier to verify the accuracy of the cluster analysis, the study results show that the Cascade K-means cluster analysis has the highest accuracy. This study also labeled the three clusters of the cluster analysis results as high, medium, and low infection levels. Policymakers could more objectively decide which states should prioritize vaccine allocation in a vaccine shortage to prevent the epidemic from continuing to expand. It is hoped that if there is a similar epidemic in the future, relevant policymakers can use the analysis procedure of this study to determine the allocation of relevant medical resources for epidemic prevention according to the severity of infection in each state to prevent the spread of infection.

## 1. Introduction

Since the emergence of the new coronavirus COVID-19 in December 2019, it has spread through the world at a rapid rate, causing a catastrophe in the field of human public health. This virus not only affects world transportation but also causes irreparable economic and human losses. The United States, which dominates the global economic system, has failed to make timely corresponding policies for epidemic prevention [1]. Although the number of infections continues to rise, the United States seems to have failed to take effective vaccine distribution and isolation measures [2]. According to [3] who investigated vaccine allocation, it is important for a region to prioritize who receives vaccines. According to [4], reasonable vaccine distribution protocols are needed in the case of regions with unstable resources. The authors used more than 100 countries to conducted data analysis of more than 100 countries and found that problems with vaccine distribution are a main cause of the spread of influenza. In [5], authors collected the global coronavirus collective infection data, used the cluster technique to analyze, and found that virus transmission is related to family and community infection. The authors in [6] found data on influenza, used feature dimension reduction to extract important information from the data, and finally used classification algorithms to evaluate the outcome.

Clustering is the disassembly of a dataset from group to group, and the comparison between clusters shows whether the difference within the cluster is small and the difference between the groups is significant; the difference is measured by the distance between observations. Academic studies in all areas often use clustering to help analyze data and reach conclusions. For example, in [7], the authors detected gas leaks by monitoring mass spectrometer data and cluster analysis. Because of disputes between tourism development and natural landscape protection, various stakeholders were included in a cluster analysis, and the analysis results were divided into four groups: conservative to radical [8]. According to financial strategies of different companies [9], non-financial companies are often analyzed by clusters. Some strategies are suitable for high-tech economic industries, while others are suitable for basic industries.

Classification refers to establishing a data classification model based on known data and their category attributes, which can help predict which label the target data will be assigned. According to [10], sonar datasets are selected through the short-time Fourier transform, and then the sonar targets are classified by few-shot learning in the small sample learning method, which improves classification accuracy. In [11], a support vector machine was used to classify different hand movements according to experimental subjects’ real-time and non-real-time EMG data, and the results showed that the human muscles set were as repetitive as fingerprints or retinas. Dritsas and Trigka [12] using different machine learning techniques to predict stroke, found that ensemble machine learning was the best approach.

In the face of the shortage of vaccines during the COVID-19 pandemic, the United States did not put forward effective epidemic prevention policies in time to prevent the infection from further expanding, resulting in an increasingly serious epidemic in the United States. This study mainly uses the COVID-19 infection case indicators collected by the COVID Tracking Project in various states of the United States. Through cluster analysis, we can distinguish the severity of infection (low, medium, and high) in each state and further allocate vaccines to states with high infection rates to carry out priority epidemic prevention measures. In this study, the data mining software WEKA is used to conduct cluster analysis and classification. After the experiment, the cluster is named after confirming the classification accuracy of the confusion matrix with classification verification. This study hopes that understanding the indicators of infection cases in each state can help allocate medical resources in the future and provide a reference for decision makers in vaccine distribution.

The structure of this study is as follows. Section 2 is a preliminary description of COVID-19, medical indicators, and vaccine distribution, combined with machine learning processes, which include feature selection, clustering method, and classification verification. Section 3 describes the dataset and the experimental process. Section 4 is the result of grouping analysis and classification verification. Section 5 is the discussion, and Section 6 is the conclusion.

## 2. Preliminary

### 2.1. COVID-19

Severe Special Infectious Pneumonia (SARS-CoV-2) aka Novel Coronavirus (COVID-19) has seriously affected people’s lives worldwide, and many scholars have conducted related studies on COVID-19. A survey [13] of seriously ill patients collected many characteristics of COVID-19 symptoms, found it to be a dangerous virus, and conjectured that early pulmonary fibrosis was a substantial basis. As the epidemic became more serious, some scholars began to explore factors closely related to the death of patients. Zhou et al. [14] used univariate and multivariate logistic regression to explore factors associated with in-hospital mortality. Zheng et al. [15] analyzed the clinical characteristics of severe and non-severely ill COVID-19 patients in 13 articles with a total of 3027 patients to identify risk factors for developing severe disease or death in COVID-19 patients to predict disease progression effectively, respond to treatment early, and allocate medical resources in a better way.

### 2.2. Medical Indicators and Vaccine Allocation

According to [16], a survey of Taiwanese medical institutions found that the quality of medical care and its organization and management are highly correlated. The Taiwan Quality Indicators Project (TQIP), which collected hospital datasets from 1998 to 2004, used this dataset and a survey in the United States to conduct data envelopment analysis and found that improvements in medical quality services could reduce costs [17]. In [18], the authors analyzed common disease characteristics to improve the quality of medical care in the country and found that data analysis could help the hospital to make better decisions.

According to [19], research on vaccine distribution has studied how to stop disease transmission effectively and found that when the number of transmissions is reduced, the number of deaths can be effectively reduced. The vaccine allocation study in [20] found that 5 to 15% of people worldwide die each year from epidemics. To counter this threat, the United States mass produces a variety of vaccines. However, these vaccines have no effect on newly emergent diseases. COVID-19 required the development and production of a completely new vaccine. Then the problem of vaccine distribution had to be considered.

### 2.3. Feature Selection Techniques

In the clustering process, if there are too many variables, the problem will often become more complicated; therefore, feature selection or feature extraction becomes very important. It can reduce the dimension of variables and make the problem simpler. The following are some commonly used feature selection methods.

#### 2.3.1. Principal Component Analysis (PCA)

According to the discussion of the principal component analysis in [21], this technique is mainly aimed at finding the vector after the data projection, hoping to maximize the data variation, find the C (covariance) value, and finally get the covariance. Algorithm 1 is shown below:
**Algorithm 1** PCA based Feature Selection.**Inputs:** X = {x_1_, x_2_, ……, x_D_} // D-dimension training dataset**Outputs:** Y = {y_1_, y_2_, ……, y_d_} // lower dimensionality d-dimensional feature set where d <= D**1**Do PCA on X for dimensionality reduction**2** Compute mean of input dataset (x)’**3** Calculate the covariance matrix Cov (x)**4**Find spectral decomposition of Cov (x) and the corresponding Eigen vectors and values E_1_, E_2_, …E_D_ to get the principal components P = (x_1′_, x_2′_, ……, x_n′_) which is a subset of X.

The purpose of using PCA to extract features is to generate a new collection of dimensionally reduced features compared to the original dataset. This will convert a *D*-dimensional dataset to a new lower d-dimensional dataset where *D* <= d, as shown in Equation (1). Let X be the original dataset and xi be the individual variables in the dataset:

(1) $$Consider\;a\;D-dimensional\;dataset\;X=\left(x_{1},\;x_{2},\;x_{3},\;\ldots \ldots ,\;x_{N}\right)$$

In this study, PCA was used to reduce the dimension of the data, and the specific steps were as follows. The first step was to calculate the mean value of *X* with Equation (2), *N* = number of observations.

(2) $${\left(x\right)}^{\prime }=\frac{1}{N}\sum_{i=1}^{N}.\left(xi\right)$$

This will help standardize the data and calculate the covariance. Standardization places variables and values of data within a given range to achieve unbiased results.

The next step is computing the covariance matrix. The covariance matrix is used to identify the correlation and dependencies among the features as shown in Equation (3).

(3) $$\mathrm{Cov}\left(x\right)=1/N\sum_{i=1}^{N}.\left(xi-xi^{\prime }\right)\left(xi-xi^{\prime }\right)T$$

The last phase is spectral decomposition of the covariance matrix using eigenvectors £_1_, £_2_, ……, £_D_ and eigenvalues λ_1_, λ_2_, ……, λ_D_. This gives *Y* as shown in Equation (4). Let Y be the lower dimensional set and *yi* be the variables.

(4) $$Y=\left(y_{1},\;y_{2},\;y_{3},\;\ldots \ldots ,\;y_{P}\right)$$

such that *Y* is the lower d-dimensional dataset and has the principal components, as shown in Equation (5).

(5) $$(Y=\left(£T1\left(x-xi^{\prime }\right),£T2\left(x-xi^{\prime }\right),£T3\left(x-xi^{\prime }\right),\;\ldots \ldots ,\;£Td\left(x-xi^{\prime }\right))T\right)$$

such that for the original dataset *X*, the new dimensional representation is *Y* which has principal components.

#### 2.3.2. Information Gain

Information gain uses a feature sorting method to rank the variables in the dataset. It mainly uses an entropy principle to measure a set of randomly generated variables [22]. The information gain-based feature selection is shown in Algorithm 2:
**Algorithm 2** Information gain-based feature selection**Inputs:** Dataset D**Outputs:** Selected Features FS**1**Start**2** Initialize threshold for gain **gt****3** Initialize feature -gain map G**4**Get attributes from D into A provided c**5**for each attribute **a** in **A****6** Find gain **g****7** IF **(g > gt)** THEN**8**  Add attribute **a** and **g** to **G****9** End IF**10**End For**11**For each element in **G****12** IF feature is found useful THEN**13**  Update **FS** with the feature**14** END IF

As can be seen in Algorithm 2, the information gain-based approach finds the information gain pertaining to the importance of features in the dataset. Equations (6) and (7) are used to compute the entropy of *x* and *y*.

(6) En(x)=−∑p(x)logp(x)

(7) En(y)=−∑p(y)logp(y)

Once entropy is computed, the difference is computed to know the gain value. In fact, the gain from *x* on *y* is the reduction in entropy values and is computed using Equation (8).

(8) IG(y,x)=En(y)−En(y/x)

#### 2.3.3. Gain Ratio

Karegowda et al. [23] introduced the extension technique of information gain in which after the information gain result appears, it will branch it separately, and then find the best score from it. The information gain metric is used to select test attributes at each node of the decision tree. Let s be a set consisting of s data samples with *m* distinct classes. The expected information needed to classify a given sample is given by Equation (9).

(9) $$I\left(s\right)=-\sum_{i=1}^{m}P_{i}\log_{2}\left(P_{i}\right)$$

$P_{i}$ is the probability that an arbitrary sample belongs to class C_i_ and is estimated by s_i_/s. Attribute A has v distinct values. Let s_ij_ be the number of samples of class C_i_ in a subset S_j_. S_j_ contains those samples in S that have value a_j_ of A. The entropy information based on the partitioning into subsets by A, is shown in Equation (10).

(10) $$E\left(A\right)=-\sum_{i=1}^{m}I\left(S\right)\frac{S_{1i}+S_{2i}+\cdots S_{mi}}{s}$$

The encoding information that would be gained by branching on A is shown in Equation (11)

(11) Gain(A)=I(S)−E(A)

The gain ratio which applies normalization to information gain using a value defined is shown in Equation (12)

(12) $$SplitInfo_{A}^{\;}\left(S\right)=-\sum_{i=1}^{v}\left(\left|S_{i}\right|/\left|S\right|\right)\log2\left(\left|S_{i}\right|/\left|S\right|\right)$$

The above value represents the information generated by splitting the training dataset *S* into v partitions corresponding to v outcomes of a test on the attribute *A*. Finally, the gain ratio is defined as shown in Equation (13).

(13) $$\mathrm{Gain}\;\mathrm{Ratio}\;\left(A\right)=\frac{Gain\left(A\right)}{SplitInfo_{A}^{\;}\left(S\right)}$$

### 2.4. Cluster Analysis

Cluster analysis aims to divide samples into different groups to maximize homogeneity within each group and maximize heterogeneity between groups. This concept is similar to “intra-group homogeneity and inter-group heterogeneity” in market segmentation. There are two commonly used clustering methods introduced as follows.

#### 2.4.1. K-Means

According to [24], K-means clustering consists of two independent stages: the first stage is to select k centers, where the k value is pre-fixed, and the next stage is to bring each data object to the nearest center. Supposing that the target object is *x*, *x_i_* indicates the average of cluster *C_i_*. The criterion function is defined as shown in Equation (14).

(14) $$E=\sum_{i=1}^{k}\sum_{x\in C_{i}}{\left|x-x_{i}\right|}^{2}$$

*E* is the sum of squares of errors of all objects in the dataset. The distance of the criterion function is the Euclidean distance, which is used to determine the closest distance of each data object to the cluster center. The distance between one vector *x* = (*x*_1_, *x*_2_, … *x*_n_) and another vector *y* = (*y*_1_, *y*_2_, … *y*_n_), is the Euclidean distance $d\left(x_{i},y_{j}\right).$ It can be calculated as shown in Equation (15).

(15) d(xi,yj)=[∑i=1n(xi−yj)2]12

#### 2.4.2. Cascade K-Means

Another clustering is based on [25], in which there is a Calinski--Harabasz metric that evaluates the number of clusters, calculates the distance, and then uses the metric to decide whether to continue the cluster and find the optimal number of clusters. The matrices *WG*^{*k*}^ are square symmetric matrices of size p × p. Let *WG* denote their sum for all the clusters as shown in Equation (16).

(16) $$WG=\sum_{k=0}^{K}WG^{\left\{k\right\}}$$

The matrices *WG*^{*k*}^ represent a positive semi-definite quadratic form *Q_k_*, and their eigenvalues and their determinant are greater than or equal to 0. The within-cluster dispersion, noted as *WGSS*^{*k*}^ or *WGSS_k_*, is the trace of the scatter matrix *WG*^{*k*}^, as shown in Equation (17).

(17) $$WGSS^{\left\{k\right\}}=Tr\left(WG^{\left\{k\right\}}\right)=\sum_{i\in \;I_{k}}||M_{i}^{\left\{k\right\}}-G^{\left\{k\right\}}|{|}^{2}$$

The within-cluster dispersion is the sum of the squared distances between the observations $M_{i}^{\left\{k\right\}}$ and the barycenter *G*^{*k*}^ of the cluster. Finally, the pooled within-cluster sum of squares WGSS is the sum of the within-cluster dispersions for all the clusters as shown in Equation (18).

(18) $$WGSS=\sum_{k=0}^{K}WGSS^{\left\{k\right\}}$$

The between-group dispersion measures the dispersion of the clusters between each other. This sum is the weighted sum of the squared distances between the *G*^{*k*}^ and *G*, the weight being the number *n_k_* of elements in the cluster *C_k_*, as shown in Equation (19).

(19) $$BGSS=\sum_{k=1}^{K}n_{K}{\left|\left|G^{\left\{k\right\}}-G\right|\right|}^{2}$$

Using the notations of Equations (18) and (19), the Calinski--Harabasz metric is shown as in Equation (20).

(20) $$CH=\frac{\mathrm{BGSS}/\left(K-1\right)}{\mathrm{WGSS}/\left(N-K\right)}=\frac{N-K}{K-1}\frac{\mathrm{BGSS}}{\mathrm{WGSS}}$$

### 2.5. Classification 

Classification is a critical method of data mining. The classification concept is to learn a classification function or construct a classification model (usually called a classifier) based on existing data. The function or model can map data records in the database to one of the given categories, and apply it to data prediction. The classifier is a general term for classifying samples in data mining, including decision tree, logistic regression, naive Bayes, neural networks, and other algorithms. The following is an introduction to the two classifiers used in this study.

#### 2.5.1. Random Forest

According to [26], the generation technology of random trees evolved from decision trees. In addition, the pattern generation method of a tree can also be used without selecting the data target first. This technique is an ensemble learning algorithm that uses bagging plus random feature sampling. The random forest training algorithm applies the general bagging technique to tree learning. Given a training set X = x_1_, …, x_n_ and a target Y = y_1_, …, y_n_, the bagging method is repeated (B times) to sample from the training set with replacement and then train a tree model on these samples:

For *b* = 1, …, *B*:

1. Sample, with replacement, *n* training examples from *X*, *Y*; call these *X_b_*, *Y_b_*.
2. Train a classification or regression tree *f_b_* on *X_b_*, *Y_b_*.

After training, the prediction for the unknown sample x can be achieved by averaging the predictions of all individual regression trees on x as in Equation (21):

(21) $$\hat{f}=\frac{1}{B}\sum_{b=1}^{B}f_{b}\left(x^{\prime }\right)$$

#### 2.5.2. Neural Network

According to [27], the neural network is one of the classic technologies inspired by the human brain. human brain can process different information content because it has different neurons. The includes hundreds of millions of neurons that can connect and share. When a neuron can continuously connect to other neurons, it can trigger the brain to control the human body to complete some behaviors. This behavior is essentially a process of learning and absorbing knowledge, while new neural connections stimulate the brain to learn new actions. The process mentioned above is similar to the one used in neural networks, where neurons can be defined as one or more nodes used. The structure of neurons is an input layer, a hidden layer, and an output layer which can be shown in Equation (22), where σ() is called the activation or transfer function, N is the number of input neurons,$\;V_{ij}$ is the weights, $x_{j}$ is inputs to the input neurons, and $T_{i}^{hid}$ is the threshold terms of the hidden neurons.

(22) $$H_{i}=\sigma \left(\sum_{j=1}^{N}V_{ij}x_{j}+T_{i}^{hid}\right)$$

## 3. Material and Methods 

### 3.1. Dataset

The dataset of this study was collected from various states in the United States (The COVID Tracking Project). The dataset variables are divided into various levels. The variables marked as grade A or above indicate that the information provided by this state is relatively sufficient and complete and vice versa. There are 44 variables in the dataset, roughly divided into cases, PCR tests, antibody tests, antigen tests, hospitalizations, death outcomes, and the state metadata, as shown in Table 1.

### 3.2. Conceptual Framework

**Figure 1:**
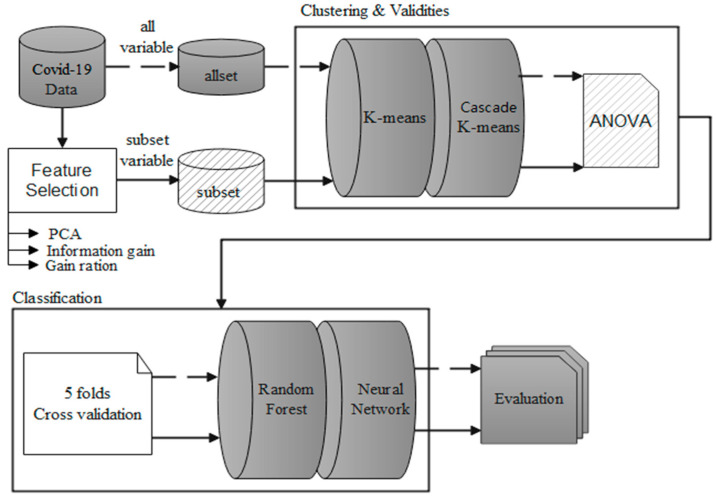
Clustering analysis and classifications scenario.

The clustering analysis and classification scenario of this study is shown in Figure 1, which are data preprocessing, cluster experiment, and classification verification. Feature selection was performed before the dataset was clustered, and essential feature variables were identified. The results of the two clustering methods were statistically compared for these essential variables. Finally, after clustering analysis, the classification method was used to verify the confusion matrix.

### 3.3. A Brief Review of Clustering Techniques

Clustering is to group all data and classify similar data into the same group. A piece of data only belongs to a particular group, and each group is called a cluster. Defining the so-called similarity is usually judged by the distance between data points. The closer the distance is, the more similar it is presumed to be. The denser the neighbors, the more similar they are presumed to be. The clustering techniques are pretty diverse. In the 1970s, most of the published studies were performed with hierarchical-based algorithms. In addition to generating a tree graph, these algorithms can also present the division of relatively important and target clusters [28]. However, when the merge or split decision is implemented in the pure hierarchical clustering method, the quality of the clustering will be affected, and it will not undo previous operations. Moreover, an object cannot move to another cluster [29]. The density-based algorithm plays a vital role in nonlinear shapes and structures derived from density. The concepts of the density-based algorithm are density accessibility and density connectivity. However, most of the indicators used to evaluate or compare cluster analysis results are not suitable for evaluating the results of density-based clustering analysis [30].

In the past few years, there has been much work on graph-based clustering, and theorists have extensively studied the properties of clustering and the quality measures of various clustering algorithms using elegant mathematical structures established in graph theory [31]. For example, the Markov Cluster Algorithm is a fast and scalable graph (also known as a network) unsupervised clustering algorithm based on the simulation of (random) flow in a graph [32]. A comparison table of different clustering techniques is shown in Table 2. The pros and cons of different clustering techniques are also included. This study employs a partitioning algorithm, a non-hierarchical approach, to evaluate clustering results by constructing various partitions. Criteria are globally optimal or efficient heuristics. K-means is the most commonly used method [33], which needs to define the number of clusters in advance to meet the requirements of specific clusters. The dataset in this study does not belong to a complex real network or shape, so the cluster analysis in this study is performed by well-integrated K-means and modified Cascade K-means.

### 3.4. Data Preprocessing

#### 3.4.1. Feature Selection

**Figure 2:**
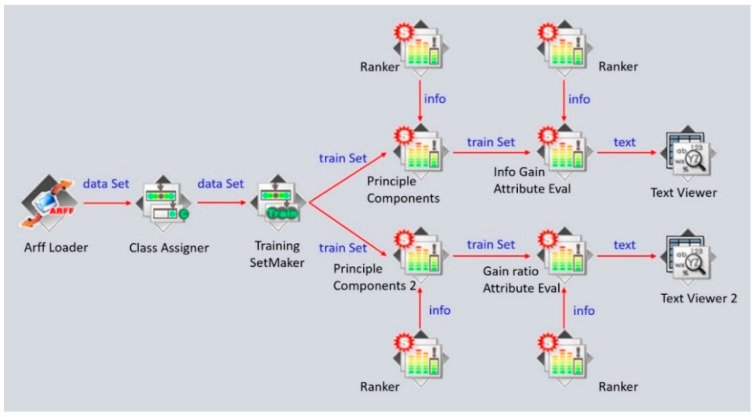
WEKA Feature selection process.

According to [34], when the data are more complex, the readability is lower; therefore the reduction of the data dimension becomes a matter of course, and the reduction of the data dimension is also a method usually used for feature selection. Uğuz [35] used the method of data dimension reduction to extract features in different mixed models using two-stage testing and finally obtained better results. Therefore, this study used PCA, IG, and GR to select essential variables and then sorted out co-occurring variables. The method of feature selection and the detailed WEKA feature selection process is shown in Figure 2. The selected common variables are used for the next step of cluster analysis.

#### 3.4.2. Clustering Analysis and Classification

**Figure 3:**
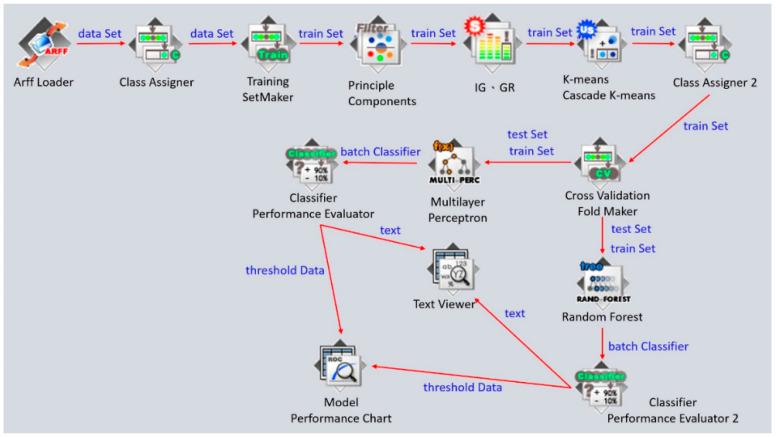
Clustering and classification validation with WEKA.

This study mainly used the dataset from 2020 to 2021 in the COVID Tracking Project to conduct cluster analysis. In the experiment, fearing that the single clustering method was too subjective, we intentionally compared the clustering results of the two clustering methods after data preprocessing. Both K-means and Cascade K-means were used in the cluster experiments in this study. The classification verification after the cluster analysis was completed. Previous research found that the random forest method (RF) and the neural network (NN) have good performance [36]. We therefore used these two methods to compare the confusion matrix in the classification results and verify the effect of cluster analysis. The detailed WEKA cluster experiment and classification verification process are shown in Figure 3.

## 4. Results

### 4.1. Feature Selection and Clustering Analysis Result

The descriptive statistics such as the minimum, maximum, average, and standard deviation of the data fields marked as grade A in The COVID tracking project dataset are shown in Table 3.

We first performed principal component analysis (PCA) on the dataset and extracted the first 20% of the variables from the PCA results. The detailed results are shown in Table 4.

Next, we continued to extract the feature variables from the IG and GR methods. Feature variable selection of the IG and GR methods also takes the top 20% of the feature variables and the top 20% of the PCA feature variables for sorting and comparison. The results are shown in Table 4. The feature variables that repeatedly appear in Table 4 are selected in this study. We list these important feature variables that repeatedly appear in Table 5. Table 6 is the cluster feature variables selected for this study, and there are 10 variables in total.

According to the critical cluster characteristics in Table 6, the number of confirmed cases, the number of deaths, the number of hospitalizations, the number of PCR tests, and other variables are related. It can be seen from this information that the results of future cluster analysis will have a positive relationship with the severity of the confirmed outbreak, and age, hospitalization, and death are closely related [37].

This study next carried out cluster analysis. The variables after feature selection (FS) in Table 5 were analyzed by K-means and Cascade K-means methods of WEKA. The results of the cluster analysis of US states are shown in Table 7.

It can be observed from Table 6 that the clustering results of these two types of cluster analysis are not the same. 

To determine which cluster analysis results were better, we first performed an ANOVA analysis of variance between clusters. Table 7 shows the results of the analysis of variance (ANOVA) between clusters after using K-means for cluster analysis. Table 8 shows that 7 of the 10 selected variables are significant. Moreover, Table 9 shows the results of ANOVA analysis between clusters after using Cascade K-means for cluster analysis. Table 9 shows that all 10 variables are significant. Therefore, the preliminary analysis results show that there are significant differences between clusters using Cascade K-means cluster analysis results, and the effect of clustering is better.

### 4.2. Classification Validation

Although the cluster analysis results verify that the clustering effect of Cascade K-means is better, we still need more evidence to prove its clustering effect. Therefore, we used two classifiers to train and test their classification effects in this section. The accuracy of the confusion matrix after classification by the classifiers can represent the clustering effect after cluster analysis. The higher the accuracy is, the better the clustering effect.

#### 4.2.1. Validation of Random Forest

This section verifies the clustering results of K-means through the confusion matrix of the random forest classifier. As shown in Table 7, the clustering results of K-means are divided into three groups, the first group has 5 states, the second group has 5 states, and the third group has 41 states. This study uses WEKA’s random forest (RF) classifier for training and testing. The confusion matrix of the test results is shown in Table 10. In the classification of the first group, a total of five states were misclassified to the third group, and four states of the second group were misclassified to the third group. The accuracy of random forest classification using the clustering results of K-means was 82.35%.

In addition, as can be seen from Table 7, the cluster analysis results of Cascade K-means are also divided into three groups. The first group has 22 states, the second group has 22 states, and the third group has 7 states. This study still uses WEKA’s random forest (RF) classifier for training and testing. The confusion matrix of the test results is shown in Table 11. In the classification of the first group, only one state was misclassified to the third group, and the rest were classified correctly, with a classification accuracy rate of 98.03%. This study again shows that the cluster analysis results of Cascade K-means are better than the cluster analysis results of K-means.

#### 4.2.2. Validation of Neural Network

In case the results of random forest are too subjective, in this section we will use another neural network classifier to verify the results of cluster analysis again. Table 12 is the confusion matrix verified by the K-means cluster analysis results through neural networks classification. As can be seen from Table 12, five states were misclassified to the third group in the classification of the first group. In addition, the second group had three states misclassified, and the third group had two states misclassified from the second group, and the classification accuracy for K-means clustering was 80.39%.

Table 13 is the confusion matrix verified by the Cascade K-means cluster analysis results through neural networks’ classification. It can be seen from Table 13 that under the classification and verification of the neural network method, there were three states in the first group and the second group each with three misclassifications, and the classification accuracy rate is 88.23%. Table 14 shows the comparison of classification verification of the two clustering methods, and it can be seen that Cascade K-means has a better clustering result in terms of accuracy, precision, and recall. The study results again show that the cluster analysis results of Cascade K-means are still better than the cluster analysis results of K-means.

The above study results show that the cluster analysis results of Cascade K-means are the best, so the following discussions are based on the cluster analysis results of Cascade K-means for more in-depth discussions.

## 5. Discussion

**Figure 4:**
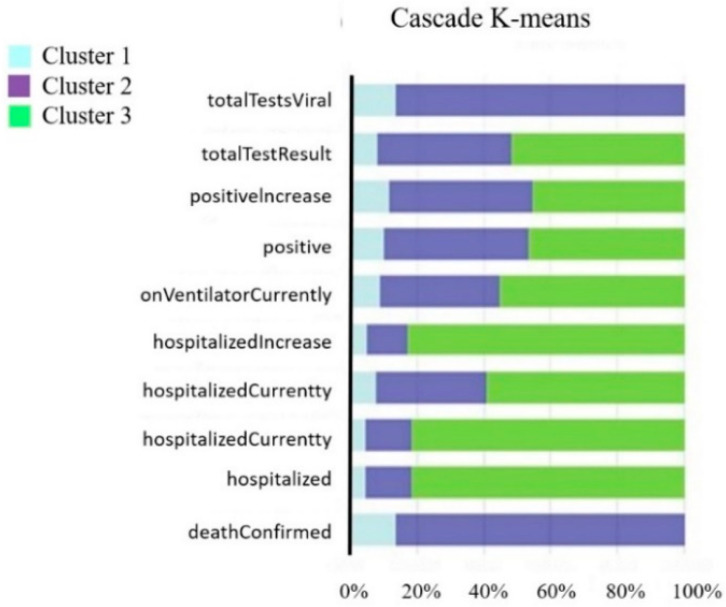
Cluster characteristics.

After classification and verification, this study uses the cluster analysis results of Cascade K-means with the highest accuracy as the cluster basis. Figure 4 shows the characteristics of the 10 feature variables of the 3 clusters. The proportion of variables in the first cluster is all below 20%, The second cluster of variables is mainly characterized by (total TestResults) and (deaths Confirmed). The third cluster of variables is mainly characterized by hospitalization-related from (hospitalizedIncrease), (hospitalizedCurrently), and (hospitalizedCumulative). Next, we will label the three clusters of the cluster analysis results. In this study, the average of the 10 feature variables of the 3 clusters was used to label the high, medium, and low severity of the epidemic infection in each state in the United States. Table 15 is the cluster labeling result of this study.

**Figure 5:**
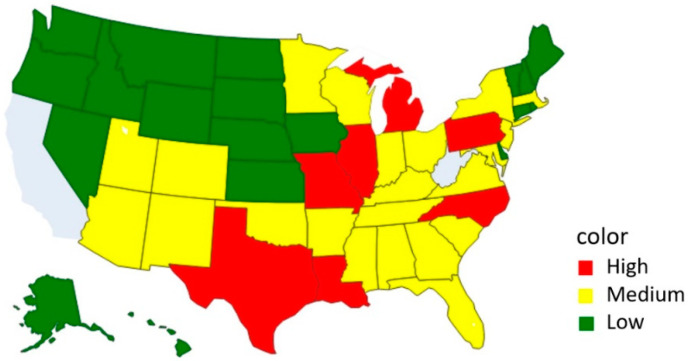
Clustering results from Cascade K-means.

**Figure 6:**
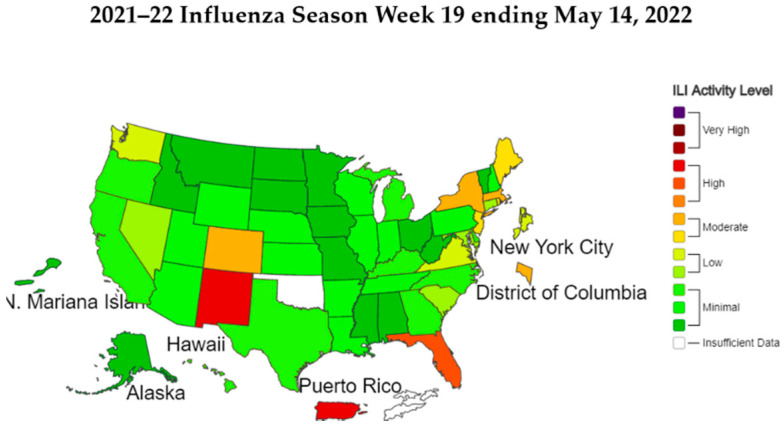
Influenza activity according to the CDC.

Nevertheless, Figure 5 is a color map of the severity of the outbreak in each US state based on the findings of this study. California in the lower-left corner was omitted because the data collected in California at the beginning of the outbreak were not sufficient and were not listed as A-level. From the color map, it can be seen that the southeastern United States was more serious. At the same time, the United States also had a large outbreak of influenza in 2021, as shown in Figure 6, and the southeastern United States was also the main infection area. That as an interesting finding. In the future, decision makers can refer to these two graphs and deduce an epidemic prevention strategy after comparing the severity to improve future epidemic prevention efficacy.

COVID-related vaccine supplies increased throughout 2021, but it will take months to get enough vaccines for everyone who needs them; existing vaccines must be distributed to priority groups until there is a sufficient supply for all [37]. In this study, the clustering variables include the number of confirmed cases, the number of deaths, PCR tests used, and the number of respirators. In addition to determining possible priorities through the clustering results, other external information can also be used to coordinate vaccine allocation. for example, to older age groups, front-line workers, or vaccine distribution models [38,39]. Wingert et al. [40] used machine learning methods to establish a cluster analysis of regional severity, which may provide an alternative perspective for future vaccine allocation by using multivariate analysis of the severity of risk factors to allocate vaccine.

## 6. Conclusions

COVID-19 has spread and ravaged worldwide since December 2019, but in the case of vaccine shortage, how to distribute vaccines is a critical issue. This study uses machine learning technique combined with the medical indicators of the COVID Tracking Project to perform a cluster analysis of various states in the United States to distinguish the severity of COVID-19 infection. The dataset of this study was collected from 2020 to 2021. After features selection, and clustering methods, and then through the classification method to verify the data, the verification results show that the clustering results of Cascade K-means are better, and the classification accuracy is the highest. This study also marked the three clusters of the analysis results of US states as high, medium, and low infection so that policymakers can better objectively decide which states should prioritize vaccine allocation to prevent the epidemic from continuing to expand in the event of a vaccine shortage. It is hoped that, if there is a similar disease pandemic in the future, relevant policymakers can use the procedure of this study to allocate relevant medical resources according to the severity of infection in each state to prevent the spread of infection and the loss of more lives. The clustering results from this study can also be combined with other external information to shape a more careful vaccine distribution plan, helping back decision makers in the event of a potential future outbreak.

## Figures and Tables

**Table 1:** Dataset.

Item	Variables	Attribute	Item	Variables	Attribute
1	date	String	24	inlcucumulative	Numerical
2	state	String	25	inlcuCurrently	Numerical
3	dataQualityGrade	String	26	onVentilatorCumulative	Numerical
4	positive	Numerical	27	onVentilatorCurrently	Numerical
5	positive Increase	Numerical	28	death	Numerical
6	probable Cases	Numerical	29	death Increase	Numerical
7	positiveScore	Numerical	30	death Probable	Numerical
8	positiveCasesViral	Numerical	31	death Confirmed	Numerical
9	positiveTestsViral	Numerical	32	recovered	Numerical
10	positiveTestsPeopleAntibody	Numerical	33	totaltestResults	Numerical
11	positiveTestsAntibody	Numerical	34	totalTestResultsIncrease	Numerical
12	positiveTestsPeopleAntigen	Numerical	35	totalTestsViral	Numerical
13	positiveTestsAntigen	Numerical	36	totalTestsViralIncrease	Numerical
14	negative	Numerical	37	totalTestsPeopleViral	Numerical
15	negativeTestsViral	Numerical	38	totalTestsPeopleViralIncrease	Numerical
16	negativeTestsPeopleAntibody	Numerical	39	totalTestEncountersViral	Numerical
17	negativeTestsAntibody	Numerical	40	totalTestEncountersViralIncrease	Numerical
18	negativeIncrease	Numerical	41	totalTestsAntigen	Numerical
19	Pending	Numerical	42	totalTestsPeopleAntigen	Numerical
20	hospitalized	Numerical	43	totalTestsAntibody	Numerical
21	hospitalized Increase	Numerical	44	totalTestsPeopleAntibody	Numerical
22	hospitalized Cumulative	Numerical			
23	hospitalized Currently	Numerical			

**Table 2:** Comparison of different clustering techniques.

Category	Hierarchical	Density-Based	Graph-Based	Partitioning
Based on	Linkage methods	Density accessibility Density connectivity	Graph theory	Mean Centroid Mediod-Centriod
Type of Data	Numerical	Numerical	Mix data	Numerical
Pros	Easy to implement Good for small datasets	Found clusters of arbitrary shapes and sizes	Perform well with complex shapes of data	Easy to implement Robust and easier to understand
Cons	Fails on larger sets Hard to find the correct number of clusters	Doe not work well in high dimensionality data.	Can be costly to compute	Unable to handle noisy data and outliers

**Table 3:** Dataset descriptive statistics.

Data Field	Minimum	Maximum	Mean	Standard Deviation
Death	3.563	20,146.993	3012.726	4063.720
deathConfirmed	0.000	11,873.819	1612.264	2564.015
deathIncrease	0.229	139.083	23.802	28.186
deathProbable	0.000	1557.983	116.001	241.290
Hospitalized	0.000	74,908.536	6795.447	12,375.868
hospitalizedCumulative	0.000	74,908.536	6795.447	12,375.868
hospitalizedCurrently	0.000	5706.697	1016.505	1178.133
hospitalizedIncrease	−0.868	574.361	52.537	93.318
inIcuCumulative	0.000	4167.848	374.140	935.623
inIcuCurrently	0.000	1594.500	198.782	310.440
negative	3193.583	5,676,868.213	1,217,574.982	1,393,361.630
negativeIncrease	225.347	66,790.590	10,430.280	13,289.475
negativeTestsAntibody	0.000	274,785.535	9939.138	42,390.124
negativeTestsPeopleAntibody	0.000	307,446.436	9400.504	44,786.195
negativeTestsViral	0.000	5,069,123.190	376,163.876	913,586.361
onVentilatorCumulative	0.000	1210.757	44.743	189.114
onVentilatorCurrently	0.000	383.805	77.040	115.523
positive	290.354	689,808.865	126,025.819	137,216.541
positiveCasesViral	0.000	644,108.814	99,837.740	122,787.805
positiveIncrease	16.833	6633.789	1390.662	1405.567
positiveScore	0.000	0.000	0.000	0.000
positiveTestsAntibody	0.000	32,553.559	2128.500	6739.150
positiveTestsAntigen	0.000	28,745.608	1748.258	5172.510
positiveTestsPeopleAntibody	0.000	30,843.331	969.265	4476.315
positiveTestsPeopleAntigen	0.000	19,372.812	912.666	3418.733
positiveTestsViral	0.000	746,688.084	69,991.507	149,301.079
recovered	0.000	548,376.917	56,375.980	91,104.014
totalTestEncountersViral	0.000	6,252,107.282	436,938.962	1,266,757.976
totalTestEncountersViralIncrease	0.000	70,634.798	4496.100	13,115.623
totalTestResults	3643.785	6,252,107.282	1,575,543.098	1,650,858.474
totalTestResultsIncrease	250.514	70,634.798	14,555.709	15,895.585
totalTestsAntibody	0.000	336,182.488	33,358.436	81,366.549
totalTestsAntigen	0.000	329,705.523	23,679.767	57,553.140
totalTestsPeopleAntibody	0.000	338,396.716	13,807.533	51,122.147
totalTestsPeopleAntigen	0.000	121,896.261	6203.051	21,180.165
totalTestsPeopleViral	0.000	3,939,157.669	424,758.638	718,630.307
totalTestsPeopleViralIncrease	−251.653	31,530.365	3374.921	5665.766
totalTestsViral	0.000	5,972,478.403	1,244,215.985	1,591,549.099
totalTestsViralIncrease	0.000	52,143.061	11,304.525	14,366.747

**Table 4:** PCA feature selection results.

Features											
Group	Pc1	Pc2	Pc3	Pc4	Pc5	Pc6	Pc7	Pc8	Pc9	Pc10	Pc11
Variation	15.89	6.05	4.69	2.64	1.90	1.38	1.11	0.92	0.64	0.54	0.49
Variation Percentage	0.42	0.16	0.12	0.07	0.05	0.04	0.03	0.02	0.02	0.01	0.01
Cumulative contribution ratio	0.42	0.58	0.70	0.77	0.82	0.85	0.88	0.91	0.93	0.94	0.96

**Table 5:** Feature selection sorting.

Rank	PCA	IG	GR	Average Rank
1	A30	A2	A39	2.7302
2	A19	A19	A11	2.7302
3	A21	A30	A13	2.7302
4	A31	A31	A16	2.7302
5	A8	A13	A18	2.7302
6	A15	A21	A19	2.7302
7	A24	A12	A38	2.7302
8	A34	A4	A12	2.7302
9	A14	A8	A9	2.7302
10	A36	A27	A22	2.6814
11	A9	A38	A8	2.4945
12	A33	A39	A3	2.4945
13	A6	A20	A4	2.4945
14	A7	A7	A5	2.3984
15	A23	A6	A6	2.3984
16	A3	A11	A7	2.3269
17	A5	A9	A21	2.3269
18	A39	A36	A20	2.2745
19	A29	A18	A2	1.9183
20	A18	A3	A31	1.9183

**Table 6:** Clustering variable.

Code	Variable	Definition
A3	deathConfirmed	Number of confirmed deaths
A6	Hospitalized	Number of hospitalizations
A7	hospitalizedCumulative	Cumulative hospitalizations
A8	hospitalizedCurrently	Number of people currently hospitalized
A9	hospitalizedIncrease	New hospitalizations
A18	onVentilatorCurrently	Number of respirators currently in use
A19	positive	Number of confirmed cases
A21	positiveIncrease	The number of new diagnoses
A31	totalTestResults	Total number of tests
A39	totalTestsViralIncrease	Number of new PCR tests

**Table 7:** Clustering results.

Method	FS + K-Means Clustering			FS + Cascade K-Means Clustering		
Group	Cluster1	Cluster2	Cluster3	Cluster3	Cluster1	Cluster2
Count	5 states	5 states	41 states	7 states	22 states	22 states
cluster member	LA MO NC PA TN	IL MA MI OH TX	AK, AL, AR, AZ, CO, CT, DC, DE, FL, GA, GU, HI, IA, ID, IN, KS, KY, MD, ME, MN, MS, MT, ND, NE, NH, NJ, NM, NV, NY, OK, OR, PR, RI, SC, SD, UT, VA, VT, WA, WI, WY	IL LA MI MO NC PA TX	AL, AR, AZ, CO, FL, GA, IN, KY, MA, MD, MN, MS, NJ, NM, NY, OH, OK, SC, TN, UT, VA, WI	AK, CT, DC, DE, GU, HI, IA, ID, KS, ME, MT, ND, NE, NH, NV, OR, PR, RI, SD, VT, WA, WY

**Table 8:** ANOVA analysis results with K-means clustering.

Source	Sum of Squares	df	*p*-Value
deathConfirmed	96,309,899.004	2	0.000 ***
hospitalized	163,810,675.372	2	0.595
hospitalizedCumulative	163,810,675.372	2	0.595
hospitalizedCurrently	20,454,751.196	2	0.000 ***
hospitalizedIncrease	10,452.301	2	0.558
onVentilatorCurrently	171,650.041	2	0.001 ***
positive	297,933,527,997.266	2	0.000 ***
positiveIncrease	33,214,641.966	2	0.000 ***
totalTestResults	56,194,090,472,628.98	2	0.000 ***
totalTestsViral	73,640,463,254,532.75	2	0.000 ***

*** *p* < 0.001.

**Table 9:** ANOVA analysis results with Cascade K-means clustering.

Source	Sum of Squares	df	*p*-Value
deathConfirmed	92,467,765.941	2	0.000 ***
hospitalized	2,542,735,930.524	2	0.000 ***
hospitalizedCumulative	2,542,735,930.524	2	0.000 ***
hospitalizedCurrently	28,731,039.605	2	0.000 ***
hospitalizedIncrease	146,195.267	2	0.000 ***
onVentilatorCurrently	182,801.937	2	0.000 ***
positive	428,233,021,802.748	2	0.000 ***
positiveIncrease	47,433,754.861	2	0.000 ***
totalTestResults	62,516,522,292,791.39	2	0.000 ***
totalTestsViral	53,777,136,225,256.51	2	0.000 ***

*** *p* < 0.001.

**Table 10:** Random forest classification validation for K-means clustering.

Confusion Matrix			Clustering Class		
			Cluster1	Cluster2	Cluster3
			a	b	c
Prediction Class	Cluster1	a	05	0	5
	Cluster2	b	0	15	4
	Cluster3	c	0	0	4141

**Table 11:** Random forest classification validation for Cascade K-means clustering.

Confusion Matrix			Clustering Class		
			Cluster1	Cluster2	Cluster3
			a	b	c
Prediction Class	Cluster1	a	2122	1	0
	Cluster2	b	0	2222	0
	Cluster3	c	0	0	77

**Table 12:** Neural network classification validation for K-means clustering.

Confusion Matrix			Clustering Class		
			Cluster1	Cluster2	Cluster3
			a	b	c
Prediction Class	Cluster1	a	05	0	5
	Cluster2	b	2	25	1
	Cluster3	c	0	2	3941

**Table 13:** Neural network classification validation for Cascade K-means clustering.

Confusion Matrix			Clustering Class		
			Cluster1	Cluster2	Cluster3
			a	b	c
Prediction Class	Cluster1	a	1922	3	0
	Cluster2	b	0	2222	0
	Cluster3	c	3	0	47

**Table 14:** Comprehensive comparison of two clustering methods.

Clustering	K-Means		Cascade K-Means	
Validation	RF	NN	RF	NN
Accuracy	0.8235	0.8039	0.9803	0.8823
Precision	1	0.911	1	0.938
Recall	0.824	0.872	0.98	0.938

**Table 15:** Cluster labeling.

Severity	States
Low	AK, CT, DC, DE, GU, HI, IA, ID, KS, ME, MT, ND, NE, NH, NV, OR, PR, RI, SD, VT, WA, WY
Medium	AL, AR, AZ, CO, FL, GA, IN, KY, MA, MD, MN, MS, NJ, NM, NY, OH, OK, SC, TN, UT, VA, WI
High	IL, LA, MI, MO, NC, PA, TX

## Data Availability

Not applicable.
